# Effect of High versus Low Dairy Consumption on the Gut Microbiome: Results of a Randomized, Cross-Over Study

**DOI:** 10.3390/nu12072129

**Published:** 2020-07-17

**Authors:** J. Casper Swarte, Coby Eelderink, Rianne M. Douwes, M. Yusof Said, Shixian Hu, Adrian Post, Ralf Westerhuis, Stephan J.L. Bakker, Hermie J.M. Harmsen

**Affiliations:** 1 Department of Nephrology, University Medical Center Groningen, University of Groningen, 9713 GZ Groningen, The Netherlands; c.eelderink@umcg.nl (C.E.); r.m.douwes@umcg.nl (R.M.D); m.y.said@umcg.nl (M.Y.S.); a.post01@umcg.nl (A.P.); s.j.l.bakker@umcg.nl (S.J.L.B.); 2 Department of Gastroenterology and Hepatology, University Medical Center Groningen, University of Groningen, 9713 GZ Groningen, The Netherlands; s.hu01@umcg.nl; 3 Department of Genetics, University Medical Center Groningen, University of Groningen, 9713 GZ Groningen, The Netherlands; 4 Dialyses Center Groningen, 9713 GZ Groningen, The Netherlands; r.westerhuis@umcg.nl; 5 Department of Medical Microbiology, University Medical Center Groningen, University of Groningen, 9713 GZ Groningen, The Netherlands; h.j.m.harmsen@umcg.nl

**Keywords:** dairy, gut microbiome, Faecalibacterium prausnitzii, Streptococcus thermophilus, constipation

## Abstract

The influence of dairy on the gut microbiome has not been studied extensively. We performed a randomized cross-over study to analyze the effect of high dairy intake on the gut microbiome. Subjects were randomly assigned to a high-dairy diet (HDD) (5–6 dairy portions per day) and a low-dairy diet (LDD) (≤1 dairy portion per day) for 6 weeks with a washout period of 4 weeks in between both diets. The gut microbiome was assessed using 16S rRNA gene sequencing. Compositionality and functionality of the gut microbiome was assessed using Quantitative Insights Into Microbial Ecology (QIIME) and Phylogenetic Investigation of Communities by Reconstruction of Unobserved States (PICRUSt). Stool consistency was evaluated using the Bristol stool chart. In total, 46 healthy overweight subjects (BMI range 25–30 kg/m^2^) completed both intervention periods. During the HDD, there was a significantly higher abundance of the genera *Streptococcus*, *Leuconostoc*, and *Lactococcus*, and the species *Streptococcus* *thermophilus*, *Erysipelatoclostridium* *ramosum* and *Leuconostoc* *mesenteroides* (*p*_FDR_ < 0.10). Furthermore, during the HDD, there was a significantly lower abundance of the genera *Faecalibacterium* and *Bilophila*, and the species *Faecalibacterium* *prausnitzii*, *Clostridium* *aldenense*, *Acetivibrio* *ethanolgignens*, *Bilophila* *wadsworthia* and *Lactococcus* *lactis* (*p*_FDR_ < 0.10). There were eight subjects who became constipated during the HDD and these subjects all had a lower abundance of *F. prausnitzii*. This is the first cross-over study in which the effect of an HDD compared to an LDD on the gut microbiome has been studied. An HDD led to a significantly different composition of the gut microbiome, with a particularly lower abundance of *F. prausnitzii* and a higher abundance of *S. thermophilus*. Constipation was observed in several subjects during the HDD. Predicted metabolic pathways were not significantly altered due to an HDD.

## 1. Introduction

The gut microbiome is the largest community of microorganisms within the body. Commensal bacteria co-exist with the host without harming them. The most vital function of the gut microbiota in the distal digestive tract is the breakdown and fermentation of nutrients into short-chain fatty acids, amino acids, vitamins, and other metabolites [1]. Diet influences the composition of the gut microbiome and accounts for the high variation between individuals [2,3]. However, the effect of dairy consumption on the gut microbiome is not fully clarified.

The availability of substrates in the gut mainly depends on dietary intake. The composition of the gut microbiome is, therefore, strongly associated with long-term dietary patterns. The gut microbiome of individuals with a dietary intake based on animal protein and fat is dominated by *Bacteroides,* while a carbohydrate-based diet is dominated by *Prevotella* [4]. The gut microbiome of individuals with a vegan diet, which is completely free of meat, eggs, and dairy products, showed significantly lower levels of *Bacteroides*, *Bifidobacterium* and *Enterobacteriaceae* [5]. Even short-term changes in diet have been shown to rapidly change the gut microbiome, which occurs when subjects switch for five days between an herbivorous and a carnivorous diet: a carnivorous diet resulted in higher levels of *Alistipes*, *Bilophila* and *Bacteroides*, while an herbivorous diet led to higher levels of *Roseburia*, *Eubacterium rectale* and *Ruminoccocus bromii* [6].

Dairy intake has been shown to have beneficial effects on body composition, weight loss, and bone mineral density [7]. However, the effect of dairy intake on the composition of the gut microbiome is not fully elucidated. The addition of 250 mL whole milk to the diet of lactose malabsorbers compared to absorbers increased Actinobacteria, *Bifidobacterium*, *Anaerostipes* and *Blautia* in both groups [8]. In a parallel group, randomized trial in overweight adults, the effect of high dairy intake during an energy restriction diet on weight loss was studied: no significant differences were found in composition of the gut microbiome between subjects with a high-dairy diet (HDD) versus subjects with a low-dairy diet (LDD) [9].

Another study compared the gut microbiome of mice on a protein-based diet and mice on a milk-based diet. Levels of Bacteroidetes and *Blautia* were significantly higher, while levels of *Prevotellaceae* were lower in mice on a milk diet [10]. Fermented milk products (FMP) that contain living microbes can also alter the gut microbiome. Three different studies in humans found similar effects of FMP on the composition of the gut microbiome: consumption of FMP, containing *Bifidobacterium animalis* and *Lactobacillus acidophilus* and *Streptococcus thermophiles*, resulted in higher gut microbiome levels of *B. animalis*, *L. acidophilus* and decreased levels of *Bilophila wadsworthia* [11,12,13].

In this study, we explored the effect of high dairy consumption on the composition of the gut microbiome. A randomized cross-over design was applied to study the effect of an HDD on the composition of the gut microbiome in healthy subjects. Using 16S rRNA sequencing, we identified multiple bacterial species that were associated to dairy consumption.

## 2. Materials and Methods

### 2.1. Design and Subjects

In this study, we analyzed the composition of the gut microbiome as a secondary outcome of a randomized cross-over study regarding the effect of high dairy consumption on glucose metabolism [14]. A cross-over design was used in which subjects were subjected to an HDD for a 6 week period and a low-dairy diet (LDD) for a 6 week period, with a wash out period of 4 weeks between the diets. Fecal samples, food diaries and gut health questionnaires were collected 3 weeks after the start of a diet-period in the University Medical Center of Groningen (UMCG). Healthy, overweight males and postmenopausal females (BMI ≥25 and ≤30 kg/m^2^), aged between 45 and 65 years who were low-medium dairy consumers were eligible for the study. Volunteers with diabetes mellitus (based on fasting plasma glucose ≥7.0 mmol/L and/or HbA1c-values >6.5% at screening), clinically relevant abnormalities in laboratory measurements of blood lipids (total cholesterol >8 mmol/L, triglycerides >6 mmol/L, or low density lipoprotein >5.7 mmol/L), hematological abnormalities (hemoglobin level <8.7 mmol/L), liver function abnormalities (aspartate aminotransferase (ASAT), alanine aminotransferase (ALAT)) and/or renal failure (estimated glomerular filtration rate (eGFR)) were excluded. Furthermore, subjects with a positive HIV, hepatitis B surface antigen (HbsAg), and/or hepatitis C at screening were excluded. Subjects with gastrointestinal disorders or subjects who had undergone digestive tract surgery (except appendectomy) were excluded. Further exclusion criteria were intake of nutritional supplements, use of medication that would interfere with the study parameters (oral anti-diabetics, insulin, lipid-lowering drugs, and antibiotics), reported weight loss, or medically prescribed diet, reported vegan, vegetarian, or macrobiotic life-style, or a reported food allergy or sensitivity (e.g., lactose-intolerance). The study was performed in adherence to the Declaration of Helsinki (version 2013) and in accordance with the Medical Research Involving Human Subjects Act (Dutch WMO). The research protocol for this study was approved by the independent ethics committee of UMCG (METc 2014/298). All subjects provided a written informed consent for the study (randomized controlled trials registration number: NTR4899)**.**

### 2.2. Dietary Intervention

One dairy portion consisted of 250 mL of semi-skimmed milk, 250 mL of buttermilk, 200 g of semi-skimmed yoghurt, or 30 g of low-fat cheese (Friesland Campina). Male subjects in the HDD had to consume 6 portions of dairy and female subjects 5 portions of dairy per day. A minimum of two portions of yoghurt and one portion of cheese had to be consumed. During the LDD, only one portion of dairy was allowed daily. Furthermore, total caloric intake was maintained that was similar to the habitual diet of the subject to maintain a stable body weight. Subjects were instructed by a dietician in the UMCG. More details about the diet, instructions and randomization process are reported in the study by Eelderink et al. [14].

### 2.3. Questionnaires

The questionnaires were collected in the 3rd week after the start of an intervention-period. Subjects were asked to fill in their total dietary consumption for 3 consecutive days (2 weekdays, 1 weekend day) in a food diary. A questionnaire was collected about intestinal health including a 7-day diary consisting of the Bristol stool chart. The Bristol stool chart is a tool designed to classify the consistency and form of human feces [15]. A score on the Bristol stool chart of 1 was considered as severe constipation and a score of 2 was regarded as mild constipation. A score of 3–5 as normal consistency. Furthermore, a score of 6 or 7 was considered as mild or severe diarrhea.

### 2.4. Body Composition

Body weight, height, waist circumference and hip circumference were measured at baseline and after 3 weeks of each test period. All measurements were performed in duplicate.

### 2.5. Fecal Samples and 16S rRNA Gene Sequence Analysis

Fecal samples of 46 healthy volunteers were collected and frozen directly at home, transported on ice and stored until analysis at a temperature of −80 °C. DNA was extracted from 0.25 g of fecal sample with the same procedure as described by Goffau et al. [16]. The V4 and V5 regions of the 16S rRNA gene were amplified by polymerase chain reaction (PCR) using 341F and 806R primers containing a 6-nucleotide barcode and an Illumina-MiSeq adapter sequence (Illumina, San Diego, California, USA) [17]. The PCR product was purified with AMPure XP beads (Beckman Coulter, Brea, California, USA). DNA concentrations were measured with a Qubit 2.0 Fluorometer (Thermo Fisher Scientific, Waltham, Massachusetts, USA). To ensure equal library presentation for each sample, dilutions were made accordingly. The normalized DNA library was sequenced using the MiSeq Benchtop Sequencer (Illumina, San Diego, California, USA) which uses Illumina sequencing. PAired-eND Assembler for DNA sequences (PANDAseq) and Quantitative Insights Into Microbial Ecology (QIIME) were used to assign operational taxonomic units (OTU’s), readouts with a quality score lower than 0.9 were excluded. ARB was used to further assign the OTU’s on species level [18,19,20]. We used Phylogenetic Investigation of Communities by Reconstruction of Unobserved States (PICRUSt) to predict metagenome functional content from 16S rRNA marker genes [21].

### 2.6. Statistical Analysis

Statistical analyses were performed with SPSS, version 23 (IBM corp., Armonk, NY, USA) and R (R Development Core Team, 2005). Data are presented as mean ± standard deviation (SD) for normally distributed data or as median with interquartile range (IQR) for non-normally distributed data. Differences between characteristics of subjects after the HDD or the LDD were tested using a paired *t*-test or a Mann-Whitney U-test. The Shannon diversity index, a measure for the diversity of the gut microbiome, was calculated using QIIME. The microbial dissimilarity matrix (Bray-Curtis) was obtained using vegdist from the *vegan* R-package [22]. Principal coordinates were constructed and plotted with the *cmdscale* function. We used multivariate analysis by linear models 2 (MaAsLin2) to find taxa or metabolic pathways that were significantly associated to the HDD or the LDD. We used diet as a fixed effect and subject number as a random effect. We used BURRITO to visualize metabolism-related pathways [23]. Two-sided *p*-values ≤0.05 were considered statistically significant. We used the false discovery rate (FDR) to correct for multiple testing. A *p*_FDR_ ≤ 0.10 was considered statistically significant. Non-normally distributed data regarding taxa were arcsine square root transformed for depiction.

## 3. Results

### 3.1. Subject Characteristics

In this randomized cross-over study, we collected fecal samples of 46 volunteers (21 (45.7%) males, 25 (54.3%) females, aged 59.0 ± 4.3 years, height 173.7 ± 9.4 cm). The mean BMI was not significantly different after the HDD (27.9 ± 1.9 vs. 27.6 ± 2.6 kg/m^2^, *p* = 0.26). Waist circumference (95.2 ± 8.7 vs. 94.8 ± 8.7 cm, *p* = 0.25) and hip circumference (106.2 ± 5.3 vs. 106.1 ± 4.9 cm, *p* = 0.35) were not significantly different after the HDD. HDL-cholesterol was significantly different between the LDD (1.46 ± 0.38 mmol/L) and the HDD (1.40 ± 0.34 mmol/L, *p* < 0.01). Total cholesterol, LDL-cholesterol and triglycerides were not significantly different between the HDD and the LDD, *p* > 0.05 (Table 1).

### 3.2. Dietary Intake

The consumption of 5 or 6 dairy portions each day led to significant changes in the diet of the volunteers, who were instructed to keep a stable weight. Subjects on the HDD consumed 312 kcal (424%) more milk and milk products (*p* < 0.001), and 110 kcal (283%) more cheese (*p* < 0.001) compared to their intake during LDD. They also consumed 51 kcal more of cereal products during HDD (*p* = 0.035). Furthermore, they consumed significantly less bread: −37 kcal (−11%); legumes: −74 kcal (−39%); fats, oils and savory sauces: −21 kcal (−14%); and meat: −69 kcal (−30%) per day during the HDD (*p* < 0.05) (Table S1). Mean dairy consumption per week during the HDD consisted of 6.7 ± 5.2 portions of milk, 11.6 ± 3.0 portions of cheese, 5.8 ± 4.1 portions of buttermilk and 13.7 ± 1.5 portions of yoghurt (Table S2). During the HDD, participants consumed 5.41 ± 0.53 servings of dairy per week. Mean dairy consumption per week during the LDD consisted of 1.1 ± 1.4 portions of milk, 2.8 ± 2.0 portions of cheese, 0.6 ± 1.0 portions of buttermilk and 2.2 ± 1.9 portions of yoghurt (Table S2). During the LDD, participants consumed 0.94 ± 0.14 servings of dairy per week. None of the participants used a non-dairy diet during the LDD.

There were several significant changes in the intake of food components during HDD compared to the LDD. Intake of mono- and disaccharides, protein, animal-based protein, saturated fatty acids, vitamin B2, vitamin B12, calcium, potassium, magnesium, zinc, and phosphorus increased significantly in the HDD (*p* < 0.05). In contrast, intake of polysaccharides, fiber, plant-based protein and unsaturated fatty acids decreased significantly during HDD (*p* < 0.05). Total caloric intake, intake of carbohydrates, and intake of total fat did not differ significantly (Table 2).

### 3.3. Diversity Analysis

**Figure 1:**
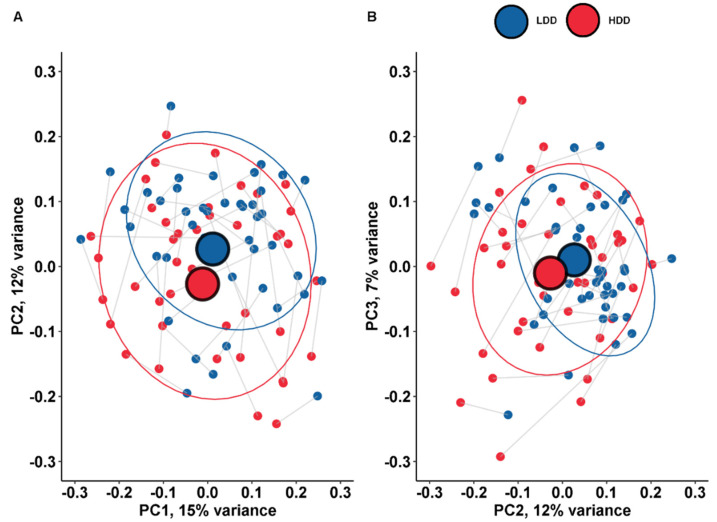
Principle coordinate analysis on the species level. The lines represent the intra-individual link of participants. This graph represents the difference between the low-dairy diet (LDD) and the high-dairy-diet (HDD) on the species level (*n* = 46). (**A**) PC1 and PC2 were not significantly different during the HDD and the LDD (*p* = 0.49 and *p* = 0.06). PC1 explained 15% of the total variance and PC2 explained 12% of variance. (**B**) PC2 and PC3 were not significantly different during the HDD and the LDD (*p* = 0.06 and *p* = 0.40). PC3 explained 7% of variance.

**Figure 2:**
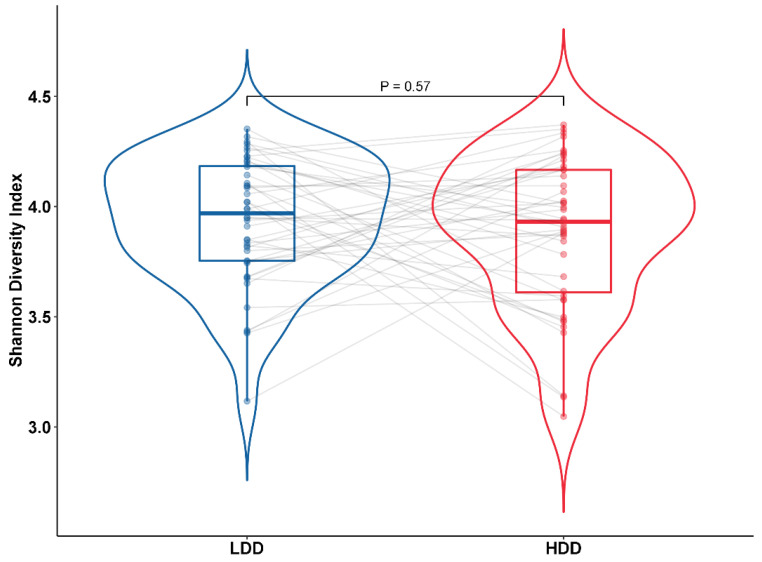
This figure depicts the Shannon diversity of the gut microbiome during the LDD and the HDD. The lines represent the intra-individual link of participants. The Shannon diversity index was not significantly different between the HDD and the LDD (paired samples Wilcoxon test, *p* = 0.57).

The taxonomic composition of microbiota on the species level of the fecal samples was visualized using principle coordinate analysis (PCoA) to show the difference between the LDD and the HDD. No major shift in gut microbiome composition was observed. There was no significant difference for principle coordinate (PC) 1, PC2 and PC3 between participants on the LDD or the HDD (Paired Wilcoxon Test, *p* > 0.05). We were unable to identify distinctive clusters in the composition of the gut microbiome on the species level when we compared the high- and low-dairy diet using the PCoA plot (Figure 1). Furthermore, we assessed the diversity of the gut microbiome using the Shannon diversity index. The median Shannon diversity index was not significantly different between subjects on the LDD (3.97, 3.75–4.18) and the HDD (3.93, 3.61–4.17) (*p* = 0.57) (Figure 2).

### 3.4. Taxonomic Composition Analysis

**Figure 3:**
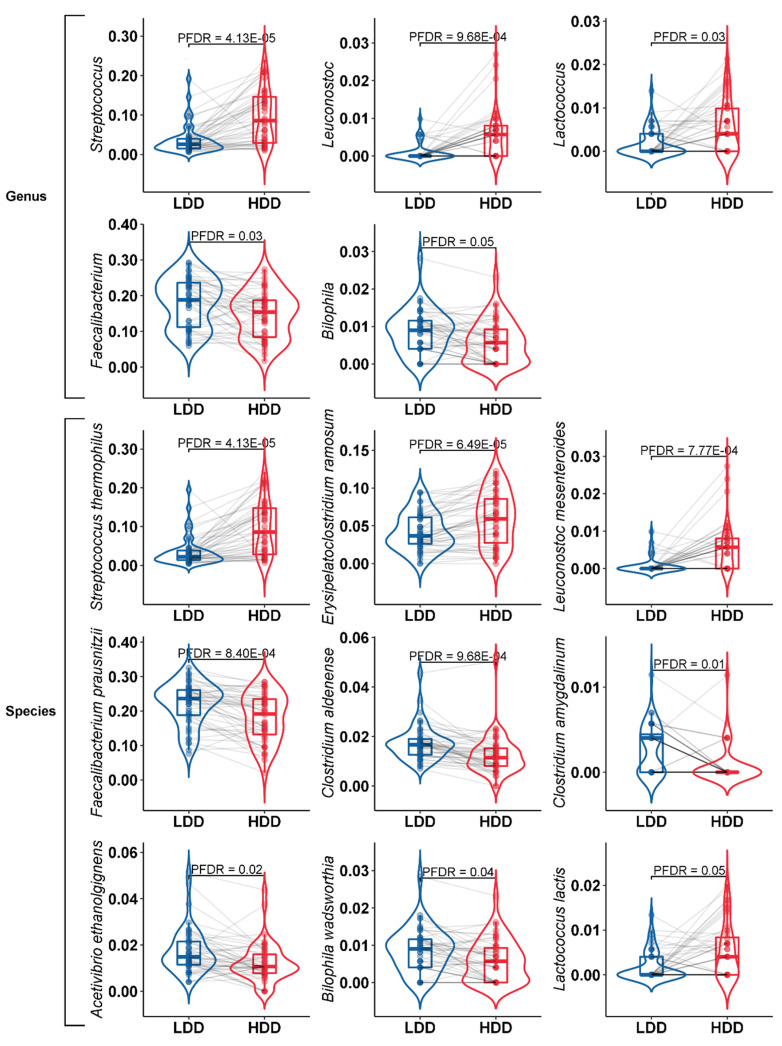
Violin plots of the relative abundance (arcsine square root transformed) of the different bacterial genera and species after the LDD and the HDD. Violin plots in blue represent the LDD and violin plots in red represent the HDD. Taxonomic level is indicated by Genus or Species.

In total, we found 24 taxa that were significantly different (*p*_FDR_ < 0.10, Table S3). Significant differences were found in the relative abundance of specific bacteria on the genus and the species level. On the genus level, the abundance of *Streptococcus*, *Leuconostoc* and *Lactococcus* were significantly increased during the HDD (*p*_FDR_ < 0.10), while the relative abundance of *Faecalibacterium* and *Bilophila* significantly decreased (*p*_FDR_ < 0.10) (Table 3) (Figure 3). On the species level, the abundance of *Streptococcus thermophilus*, *Erysipelatoclostridium ramosum* and *Leuconostoc mesenteroides* were significantly increased during the HDD compared to the LDD (*p*_FDR_ < 0.10). The abundance of *Faecalibacterium prausnitzii*, *Clostridium aldenense*, *Acetivibrio ethanolgignens*, *Bilophila wadsworthia* and *Lactococcus lactis* decreased significantly during the HDD compared to the LDD (*p*_FDR_ < 0.10) (Figure 3).

### 3.5. Predicted Metabolic Pathways

**Figure 4:**
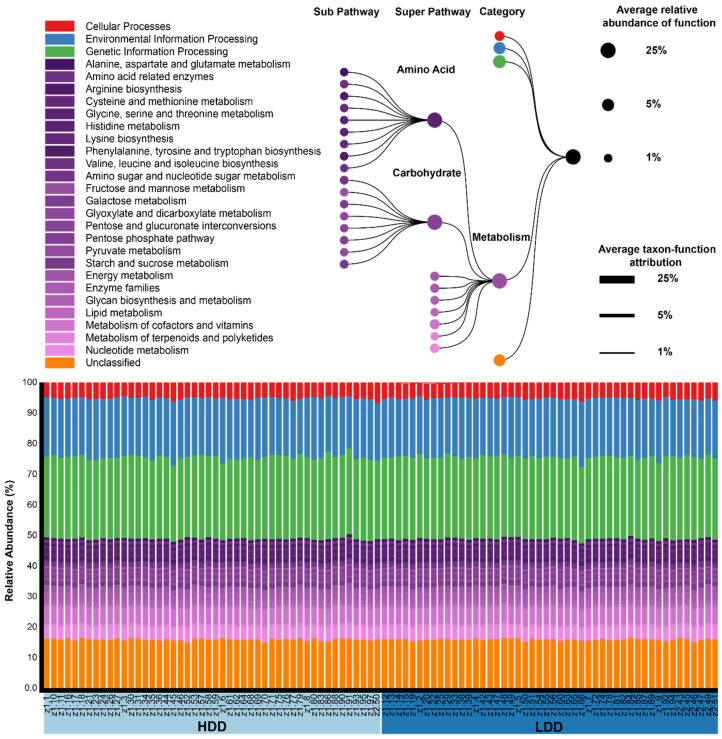
This figure depicts KEGG metabolism pathways obtained from PICRUSt and visualized with BURRITO. We highlighted the metabolism category and amino acid and carbohydrate super pathways. We found no significant differences in metabolic-related pathways (*p*_FDR_ > 0.10).

We used PICRUSt to annotate predicted gene families into KEGG Orthology (KO) and this allowed us to analyze how KEGG pathways were different between the diets. We analyzed the associations of KEGG pathways to the HDD using linear mixed models with subject number as a random effect. In total, 24 KEGG pathways were significantly different when we compared the LDD and the HDD (Table S4). We specifically focused on the metabolism category since we hypothesized that a high amount of dairy intake could potentially change metabolic-related pathways (Figure 4). At the super pathway level in the metabolism category, only metabolism of other amino acids was significantly increased during the HDD (*p*_FDR_ = 1.41 × 10^−3^). In the sub pathway level, no metabolism-related pathways were significantly different (*p*_FDR_ > 0.10).

### 3.6. Stool Consistency

**Figure 5:**
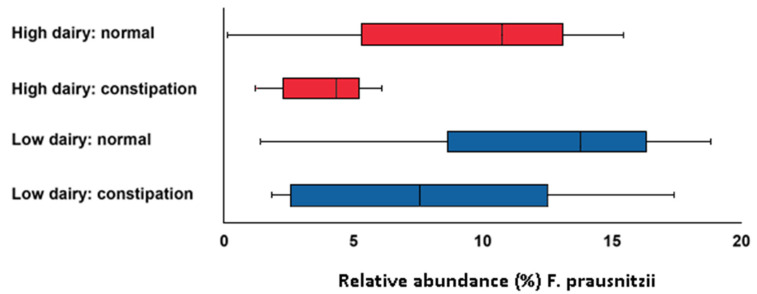
Relative abundance of *Faecalibacterium prausnitzii* for subjects with constipation (Bristol stool chart score of 1 or 2) and normal consistency (Bristol stool chart score of 3, 4 and 5) in the LDD and HDD (*p* = 0.01).

Notably, mean stool consistency score was 3.88 ± 0.81 during the LDD and 3.29 ± 1.13 during the HDD (*p* < 0.001). There were 5 subjects that developed severe constipation and 3 subjects who developed mild constipation during the HDD (Bristol stool chart score of 1 or 2). Subjects with constipation had a lower relative abundance of *F. prausnitzii* (*p* = 0.01) (Figure 5). There were no subjects with diarrhea (Bristol stool chart score 6 or 7).

## 4. Discussion

In this cross-over study, we analyzed the effect of dairy consumption on the gut microbiome composition of middle-aged, overweight subjects. We have demonstrated that a large change in dairy intake results in significant changes in the gut microbiome. Strikingly, the level of *Faecalibacterium prausnitzii* was significantly decreased during the HDD while the level of *Streptococcus thermophilus* was significantly increased compared to the LDD. There was no change in the total diversity of the gut microbiome as seen with the Shannon index.

*F. prausnitzii* is one of the most abundant bacterial species found in the gut and is associated with various beneficial functions. For instance, *F. prausnitzii* has been found to have several anti-inflammatory effects such as the ability to induce an anti-inflammatory cytokine profile [24]. Furthermore, *F. prausnitzii* plays an important role in maintaining the intestinal gut barrier function through the production of butyrate, which is an energy source for the gut epithelial cells and enhances the expression of tight junction proteins [25]. A decrease in the level of *F. prausnitzii* has been associated with inflammatory bowel disease (IBD) [26]. In the current study, an HDD resulted in decreased levels of *F. prausnitzii*, which could potentially result in a loss of the protective functions of this beneficial bacterium.

A potential explanation for the decrease in the level of *F. prausnitzii* might be a change in metabolic cross-feeding during the HDD. The majority of host energy derived from fermentation in the gut is produced in the form of short-chain fatty acids (SCFA), the major products being acetate, propionate, and butyrate. Butyrate is mainly produced by strict anaerobic species, such as *Eubacterium rectale*, *Roseburia spp.* and *F. prausnitzii* [27]. In this study, a decrease in *F. prausnitzii* was accompanied by a decrease *Roseburia faecis* in the HDD. These three bacteria can each utilize dietary fiber to form butyrate from acetyl-CoA and acetate. In contrast, lactate-producing bacteria were increased during the HDD. Lactate is produced by *S. thermophilus* and other lactic acid bacteria present to a great extent in most dairy products. Moreover, other lactate-producing bacteria, such as *Bifidobacteria*, showed a trend towards higher level in the HDD. These findings suggest less butyrate production or an alternative pathway for butyrate formation with HDD, most likely via lactate utilization as described for *Eubacterium hallii* [28]. Next to the energy function of butyrate, it also plays an important role in trans-epithelial fluid transport, reduction of inflammation and oxidative stress, reinforcement of the epithelial barrier, and it has protective properties against colorectal cancer [29]. Therefore, a decreased level of butyrate-producing bacteria will be detrimental for gut health. Whether this production is indeed lower in the HDD needs to be tested by measuring the SCFA’s in the stool. Interestingly, we found a significantly higher abundance of *S. thermophilus* in the HDD. *S. thermophilus* is present in yoghurt as a symbiotic starter for the fermentation process in milk. Subjects on the HDD consumed at least two portions of yoghurt a day. Therefore, it is likely that the increase in *S. thermophilus* is a result of higher yoghurt consumption. *S. thermophilus* is able to metabolize lactose, glucose and galactose [30], so it is likely that because of the high dairy consumption more lactose was available in the gut.

Subjects in the HDD had a significantly harder stool consistency, likely due to a significantly lower dietary fiber consumption. Moreover, eight subjects with constipation (Bristol stool chart score <3) had lower levels of *F. prausnitzii*, which may suggest a lower butyrate production. A study in rats demonstrated that butyrate enhanced colonic motility via the release of 5-hydroxytryptamine, which, in turn, stimulates the release of acetylcholine which results in colonic muscle contraction. Moreover, butyrate and propionate increase colonic muscle contractions in a dose-dependent manner [31]. Because of the lower relative abundances of *E. rectale*, *Roseburia* spp. and *F. prausnitzii*, it is possible that the constipation could be attributed to a lower motility in the colon.

This current study should be interpreted within its limitations. During the HDD, dairy intake was very high compared to normal intake, while during LDD, dairy intake was much lower. Despite this large difference, we found no notable difference in the metabolic pathways based on 16S rRNA gene analysis. However, this technique only allowed us to make predictions for the pathways based on annotated 16S rRNA genes. Future studies should use metagenomic sequencing to characterize more comprehensive metabolic pathways during a high-dairy diet. The current results should also be interpreted with notion that a large portion of the HDD consisted of fermented dairy products, which contained *S. thermophiles*, and likely explain the higher presence of this microorganism during HDD. In a next study, it would be interesting to additionally investigate the effect of non-fermented dairy products (e.g., milk, without added microorganisms) on the gut microbiome. Another limitation of the current study is that we were only able to collect one fecal sample during each intervention period. It has previously been reported that averaging time-series data improves abundance estimation [32]. Future studies should collect multiple fecal samples during the intervention periods and could measure SCFA to analyze whether a reduction in butyrate-producing bacteria leads to a reduced availability of butyrate in the gut during an HDD.

## 5. Conclusions

In conclusion, this cross-over study in which the effect of a, HDD on the gut microbiome has been studied, shows that dairy intake has substantial effects on the gut microbiome. An HDD led to a reduction of specific butyrate-producing bacteria, predominantly *F. prausnitzii,* and led to an increase in *S. thermophilus.* Predicted metabolic pathways were not significantly altered due to an HDD.

## Figures and Tables

**Table 1:** Subject characteristics.

	Low-Dairy Diet	High-Dairy Diet	*p*-Value
BMI, (kg/m^2^)	27.6 ± 2.6	27.9 ± 1.9	0.26
Body weight, (kg)	83.3 ± 11.2	84.2 ± 9.6	0.28
Waist, (cm)	94.8 ± 8.7	95.2 ± 8.7	0.25
Hip, (cm)	106.1 ± 4.9	106.2 ± 5.3	0.35
Cholesterol, (mmol/L)	5.29 ± 0.93	5.26 ± 0.77	0.68
LDL cholesterol, (mmol/L)	3.55 ± 0.81	3.54 ± 0.34	0.80
HDL cholesterol, (mmol/L)	1.46 ± 0.38	1.40 ± 0.34	<0.01
Triglycerides, (mmol/L)	1.02 (0.86–1.35)	1.10 (0.94–1.39)	0.07

**Table 2:** Food components during the high- and low-dairy diet.

	Low-Dairy Diet	High-Dairy Diet	AD ^1^	PD (%) ^2^	*p*-Value
Energy, (kcal)	2160.8 ± 525.5	2224.3 ± 429.1	63.5	3	0.32
Carbohydrates total, (g)	227.6 ± 62.1	232.7 ± 58.6	5.1	2	0.35
Mono and disaccharides total, (g)	92.0 ± 33.0	114.3 ± 31.0	22.3	24	<0.001
Polysaccharides total, (g)	134.8 ± 39.9	118.0 ± 36.8	−16.8	−12	0.001
Fiber total, (g)	22.3 ± 6.0	19.2 ± 6.0	−3.1	−14	0.002
Protein total, (g)	79.6 ± 19.3	103.2 ± 17.5	23.6	30	<0.001
Protein animal based, (g)	43.4 ± 17.2	73.2 ± 16.0	29.8	68	<0.001
Protein vegetable based, (g)	35.9 ± 11.8	29.9 ± 8.0	−6.0	−17	<0.001
Fat total, (g)	82.2 ± 25.5	80.7 ± 20.3	−1.5	−2	0.81
Fatty acid total saturated, (g)	25.0 ± 8.0	30.6 ± 6.3	5.6	22	<0.001
Fatty acid unsaturated, (g)	18.0 ± 7.5	15.0 ± 6.9	−3.0	−16	0.02
Vitamin B2, (mg)	1.4 ± 0.6	2.6 ± 0.6	1.2	89	<0.001
Vitamin B12, (µg)	4.3 ± 2.7	6.8 ± 2.2	2.5	57	<0.001
Vitamin D total, (µg)	3.4 ± 1.7	3.1 ± 1.4	−0.3	−9	0.40
Calcium, (mg)	699.4 ± 159.8	1964.4 ± 255.0	1265.0	181	<0.001
Sodium, (mg)	2612.2 ± 827.6	2583.1 ± 633.6	−29.1	−1	0.67
Potassium, (mg)	3424.9 ± 747.0	4023.8 ± 780.5	598.9	17	<0.001
Magnesium, (mg)	353.5 ± 96.6	396.8 ± 78.8	43.3	12	<0.01
Zinc, (mg)	10.2 ± 2.7	13.1 ± 2.4	2.9	28	<0.001
Phosphorus, (mg)	1316.5 ± 300.2	2012.3 ± 326.3	695.8	53	<0.001

^1^ Absolute difference (AD). ^2^ Percentage difference (PD) of the high-dairy diet (HDD) compared to the low-diary diet (LDD).

**Table 3:** The relative abundance of different bacteria on taxonomic genus level.

	Low-Dairy Diet		High-Dairy Diet				
	Median (%)	IQR (%)	Median (%)	IQR (%)	PD (%) ^1^	*p*-Value ^2^	*p*_FDR_
*Streptococcus*	0.15	(0.05–0.43)	1.81	(0.21–5.17)	1077	1.25 × 10^−7^	4.13 × 10^−5^
*Leuconostoc*	0.00	(0.00–0.00)	0.01	(0.00–0.02)		8.87 × 10^−6^	9.68 × 10^−4^
*Lactococcus*	0.00	(0.00–0.00)	0.004	(0.00–0.02)		4.84 × 10^−4^	0.03
*Faecalibacterium*	8.34	(2.80–13.14)	5.30	(1.41–8.60)	−36	4.96 × 10^−4^	0.03
*Bilophila*	0.02	(0.00–0.04)	0.01	(0.00–0.02)	−60	9.88 × 10^−4^	0.05
*Dorea*	1.48	(1.03–2.11)	1.88	(1.21–2.89)	27	0.01	0.21
*Lachnobacterium*	0.07	(0.01–0.14)	0.01	(0.00–0.06)	−91	0.01	0.23
*Parvimonas*	0.00	(0.00–0.00)	0.00	(0.00–0.004)		0.04	0.61
*Slackia*	0.04	(0.00–0.15)	0.07	(0.00–0.25)	79	0.06	0.61
*Ruminococcus*	5.02	(3.31–7.45)	4.75	(2.76–7.38)	−5	0.12	0.61
*Coprococcus*	6.29	(4.40–8.04)	5.38	(4.08–6.95)	−14	0.12	0.61
*Blautia*	9.27	(6.16–13.56)	9.79	(7.77–14.64)	6	0.17	0.61
*Dialister*	0.04	(0.00–0.88)	0.03	(0.00–0.70)	−30	0.2	0.61
*Collinsella*	1.78	(1.20–2.32)	1.86	(0.95–3.90)	5	0.23	0.61
*Eubacterium*	0.12	(0.01–1.62)	0.15	(0.01–2.02)	25	0.51	0.78
*Roseburia*	4.82	(2.60–10.13)	5.89	(1.83–10.66)	22	0.66	0.87
*Paraprevotella*	0.01	(0.00–0.07)	0.00	(0.00–0.02)	−100	0.69	0.89
*Bifidobacterium*	2.27	(0.80–5.60)	1.86	(0.54–5.17)	−18	0.69	0.89
*Bacteroides*	1.47	(0.77–3.09)	1.48	(0.61–3.13)	1	0.73	0.91
*Oscillospira*	2.04	(0.98–2.62)	1.87	(0.64–3.42)	−9	0.9	0.99

^1^ Percentage difference of the HDD compared to the LDD. ^2^ *p*-values were obtained from the Linear Mixed-Effects model.

## Supplementary Materials

The following are available online at https://www.mdpi.com/2072-6643/12/7/2129/s1, Table S1: Caloric value of food groups ingested during the high and low dairy diet, Table S2: Dairy portions consumed per week per type of dairy product, Table S3: Results of differential abundance analysis of taxonomy, Table S4: Results of differential abundance analysis of pathways.
